# On the Premature Dementia of Puberty and Adolescence

**Published:** 1903-05-30

**Authors:** W. Lloyd Andriezen

**Affiliations:** late Deputy Medical Superintendent Darenth Asylum; formerly Medical Officer and Pathologist at the West Riding Asylum


### Hospital Clinics and Medical Progress.

### ON THE PREMATURE DEMENTIA OF PUBERTY AND ADOLESCENCE.

(*Continued from page* 132).

By W. LLOYD ANDRIEZEN, M.D.Lond., late Deputy Medical Superintendent Darenth Asylum; formerly Medical Officer and Pathologist at the West Riding Asylum.

*The Varieties of Dementia Præcox.*—A study of cases seen during the past eleven years in asylums and in private practice has led me to recognise the existence of the following five varieties of dementia præcox. The classification given below agrees in the main with that which has been propounded in recent years by Kræpelin, with the addition that a "criminaloid form" of dementia præcox is accepted as worthy of inclusion in the following scheme, and that the nomenclature is slightly modified for purposes of uniformity and clearness. The five varieties of dementia præcox include the following:—

(A) *D. præcox simplex,* a simple and mild form of adolescent mental enfeeblement, with little or no mental confusion or hallucinatory excitement. References to and brief descriptions of this condition have already been given.

(B) *D. præcox hebephrenica,* a confusional form of adolescent insanity. This is often loosely named "adolescent mania" and "adolescent melancholia."

(C) *D. præcox catatonica,* a variety that is much less frequent than the preceding, a graver disorder, marked by special physical (cataleptiform) and mental symptoms.

(D) *D. præcox paranoides,* a rare variety, characterised by exuberant unsystematised delusions.

(E) *D. præcox criminalis,* a neurasthenic anti-social type, with marked criminal and parasitic tendencies of life, frequently met with in the world at large, though rare in asylums. The degree of mental alienation in this type corresponds to that of dementia præcox simplex, and there is superadded to this basis a strong taint of anti-social and criminal instincts.

The pathogenesis of dementia præcox and the significance of its division into the five groups enumerated above will be more clearly apprehended after a few preliminary considerations are taken into account. Dementia præcox, as a group of insanities, is marked by early and distinct obscuring and blunting of the intelligence supervening in puberty and adolescence. This is common to all the varieties of this class of mental affections. In the confusional (hebephrenic) form it is further characterised by the prevalence of a dream-like state compounded of mental confusion, hallucinations, and mobile delusions, and which may be interrupted by impulsive outbreaks. Indeed, delusions of slow, gradual, and systematic evolution, tending to transformation of the ego or personality, as in paranoia, do not occur in dementia præcox. The early onset of mental deterioration and enfeeblement in dementia præcox contrasts greatly with the morbidly acute sensibility and long-continued lucidity of the mental atmosphere in many paranoiacs which favour the logical and systematic evolution of delusions. Nor is the reasoning capacity of the paranoiac even approached by the apathetic and dulled brain of the subject of dementia præcox. The pathogenesis is different from the outset in these two classes of patients. Again, the so-called "adolescent melancholia" of some writers is not comparable to vesanic melancholia, or melancholia proper in its classical sense and form. The latter is an affection of the adult and developed brain, often of a finely developed but slightly unstable brain; indeed, as Clouston has pointed out many years ago, the finer the brain development the more typical and striking is the melancholia which it is capable of manifesting. The depression of spirits exhibited at times by hebephrenic or hysterical or catatonic cases of dementia præcox is not a true melancholia, but is either a shallow depression, fickle and varied by periods of liveliness, gaiety and silly acting, or a condition of apathy or clouding of consciousness—essentially a state of dementia—in which the mental atmosphere is pervaded by a persecutory mood or by mobile delusions of a persecutory and offensive character. Indeed the mood and temper of the patient is highly variable, and the phraseology which would most aptly designate the entire mental state is "mental confusion occurring on a basis of dementia." Nor, again, is the state of dementia præcox rightly named mania. The essential and underlying psychosis is a basis of dementia on which are superinduced the forms of mental confusion, etc., already alluded to. There is not in dementia præcox the quick flow and fanciful change of ideas of mania, and the quaint, whimsical and lively mood of mania proper is absent in the former. The maniac is constantly distracted in speech and act by every little object and circumstance in the environment, the attention of the adolescent dement is not thus distractable, flighty, and mobile, but is on the contrary dulled and torpid. The affective condition, moreover, in dementia præcox is variable and inconstant. By such comparisons as the above we are helped to picture to ourselves clearly what dementia præcox is and what it is not.

In the ætiology of dementia præcox the following factors may be enumerated in the order of their importance, viz.:—(*a*) Psychopathic heredity; (*b*) the disturbing influences (stresses) of puberty and adolescence on a brain afflicted with such heredity; and (*c*) debilitating causes, such as cerebral over-exertion, masturbation, and the occurrence of febrile affections, such as influenza, in childhood and during pubescence. As regards psychopathic heredity it is shown clearly from statistical data published by Kraepelin, Bevan Lewis, Christian and others that in two-thirds of all asylum cases of dementia præcox there is traceable a distinct insane, neurotic, or epileptic parentage. In the milder cases seen in practice the percentage of psychopathic heredity appears to be not different in my own collected observations (11 out of 19 cases) during the past five years. The disturbing influence of puberty and adolescence, even on the normal brain, is well known and generally recognised. On the brain which is handicapped by a psychopathic or neurotic hereditary taint the mental perturbation evoked is greater. Many cases of mild congenital imbecility are unable to pass through the period of puberty and adolescence without developing some notable amount of mental hebetude and deterioration at this critical stage, while epileptics (whether idiot, imbecile, or weak-minded) tend to undergo distinct mental deterioration and to become worse in passing through the stage of adolescence. With reference to debilitating causes I am led to regard masturbation both as an early symptom in dementia præcox, and as an aggravating cause of the stupor characteristic of many cases. As regards age and sex, dementia præcox occurs twice as frequently (or according to Kraepelin three times as frequently) in the male as in the female. The vast majority of cases (slightly over two-thirds) have their onset between the ages of 15 and 25 years, a few occur later, namely between the ages of 25 and 30 years, and a very few are of highly precocious development, appearing between the ages of 12 and 15 years. From statistical observations carefully collected and sifted (Kraepelin, Trömmer, Masselon, and personal observations), it appears that in married women the onset of dementia præcox is later than in single women. Thus in the former the age of onset is between 25 and 35 years, and in the latter between 16 and 21 years, a curious fact of which I am able to offer no adequate explanation.

*(To be Continued.)*

